# Debonding of Clear Aligner Attachments With Fluorescence-Aided Identification Technique (FIT): An *In Vitro* Study

**DOI:** 10.3290/j.jad.c_2806

**Published:** 2026-09-22

**Authors:** Inga Vanessa Bordihn, Cita Nottmeier, Henriette Ida Ulrike Stasche, Gina Marie Georgi, Ellen Schulz-Kornas, Tobias Meißner, Rainer Haak, Julian Petersen, Till Koehne

**Affiliations:** a Inga Vanessa Bordihn* Dentist, Department of Orthodontics, University of Leipzig, Leipzig, Germany. Methodology, conducted the study, performed the analysis, wrote the manuscript.; b Cita Nottmeier* Orthodontist, Department of Orthodontics, University of Leipzig, Leipzig, Germany. Performed analyses, wrote the manuscript.; c Henriette Ida Ulrike Stasche Dentist, Department of Orthodontics, University of Leipzig, Leipzig, Germany. Methodology, conducted the study, reviewed and edited the manuscript.; d Gina Marie Georgi Dentist, Department of Orthodontics, University of Leipzig, Leipzig, Germany. Methodology, conducted the study, reviewed and edited the manuscript.; e Ellen Schulz-Kornas Head of Research Department, Department of Cariology, Endodontology and Periodontology, University of Leipzig, Leipzig, Germany. Acquisition and interpretation of data, reviewed and edited the manuscript.; f Tobias Meißner Research Fellow, Department of Cariology, Endodontology and Periodontology, University of Leipzig, Leipzig, Germany. Acquisition and interpretation of data, reviewed and edited the manuscript.; g Rainer Haak Professor and Chair, Department of Cariology, Endodontology and Periodontology, University of Leipzig, Leipzig, Germany. Methodology, supervision, reviewed and edited manuscript, reviewed and edited the manuscript.; h Julian Petersen Head of Research Department, Department of Orthodontics, University of Leipzig, Leipzig, Germany. Acquisition and interpretation of data, reviewed and edited the manuscript.; i Till Koehne Professor and Chair, Department of Orthodontics, University of Leipzig, Leipzig, Germany. Methodology, Acquisition and interpretation of data, reviewed and edited the manuscript.

**Keywords:** clear aligner attachments, fluorescence-aided identification technique (FIT), composite residue, enamel loss, OCT, bovine teeth

## Abstract

**Purpose:**

The aim of this *in vitro* study was to investigate whether removing fluorescent clear aligner attachments under UV light improves the efficiency of attachment removal and reduces enamel loss and composite residue.

**Methods and Materials:**

Clear aligner attachments were bonded to bovine teeth using fluorescent-modified composite (AlignerFlow LC, VOCO, Germany); 20 models were prepared. In a split-mouth design, attachments were removed either using a fluorescence-aided identification technique (FIT) under UV light, or with standard white light (non-FIT). Removal time was recorded for each attachment. Intraoral surface and optical coherence tomography (OCT) scans (T0, T1) were analyzed to quantify enamel loss and composite residue using Avizo Software, while OCT scans were assessed using a dichotomous scale for enamel loss and a four-point scale for composite residue.

**Results:**

Although there is a trend toward faster attachment removal with FIT, measurements of removal time did not differ significantly between the groups due to high inter-individual variability. Volumetric analysis revealed a significant reduction in composite residue with FIT, while no significant differences in enamel loss were detected between the groups. OCT measurements revealed no significant differences between groups, as composite residue and enamel loss occurred on a microscopic scale in most of the specimens, regardless of the debonding method.

**Conclusion:**

While neither debonding time nor enamel loss were affected, FIT significantly reduced residual composite after attachment removal, suggesting that FIT may contribute to more standardized clinical outcomes. Future *in vivo* studies with larger sample sizes, multiple operators, and different adhesive systems are needed to validate these findings.

In recent years, orthodontic treatment has seen a notable shift in patient demographics, with a steady increase in adult patients. Since adult patients tend to have higher esthetic demands,^12^ clear aligner treatments have become an appealing treatment approach.^20,29
^ Beyond the cosmetic advantages, several studies demonstrated that aligners offer improved oral hygiene and comfort compared to conventional fixed bracket appliances.^1,22,40
^


Despite certain limitations,^22,28
^ the application of composite attachments allows for controlled and predictable tooth movements, leading to results comparable to conventional bracket therapy in patients with minor to moderate malocclusions.^17^ Whenever composite is bonded to the surface of a tooth, the question of how to safely and effectively remove it post-treatment inevitably arises. The debonding of orthodontic appliances is known to alter the enamel surface and carries the risk of iatrogenic damage. This surface damage can result either from removing excessive amounts of enamel or from incomplete elimination of remnants and adhesive residues.^24^ Such surface alterations have been associated with an increased risk of microbial colonization and subsequent enamel demineralization, highlighting the clinical importance of thorough adhesive removal.^11^


To minimize the potential damage, the fluorescence-aided identification technique (FIT) has been introduced as a method to improve the detection and removal of composite materials.^16,17
^ Fluorescence is the ability of a substance to absorb light of a shorter wavelength and emit it at a longer, visible wavelength. This effect is achieved by adding rare earth oxides to composite materials^37^ and illuminating them with ultraviolet (UV) light.^19^ Different adhesives and composites exhibit varying fluorescent properties, with discrepancies even observed between shades of the same product line.^19^ Originally, these oxides were added to restorative materials to enhance their ability to mimic natural tooth substance under different lighting conditions.^9^ By taking advantage of the fluorescent characteristics, FIT was developed to improve the clinician’s ability to distinguish composite from natural tooth structure. Studies consistently report that this approach simplifies the detection of direct restorations,^16,18
^ the removal of trauma splints^17^ and orthodontic brackets.^7,15,28,33
^


As orthodontic procedures are commonly performed on healthy teeth, preserving the integrity of the enamel surface is a primary objective when removing attachments after treatment. As attachments are often placed in esthetically relevant areas, their removal must be performed carefully without causing long-term damage or discoloration.

The present study aimed to investigate whether removing clear aligner attachments with FIT under UV light improves the efficiency of attachment removal and reduces enamel loss and composite residue. It was assumed that the use of FIT accelerates the debonding process and reduces the amount of residual adhesive and composite.

## Methods and Materials

### Specimen Preparation

The study design and experimental procedure are summarized in Figure 1. Bovine jaws were obtained from local butchers’ shops; permanent first and second incisors were extracted and then manually debrided to remove adherent soft tissues. The bovine jaws were obtained as a byproduct of the meat industry; no animals were harmed for this research. According to the regulations of the Ethics Committee of the Medical Faculty, teeth exhibiting fractures or other visible damage were excluded from this study. A total of 96 bovine incisors were included; the sample size was calculated with G*Power 3.1.9.7 based on previous studies examining FIT.^7,28,33,34
^ The teeth were stored in a 2% chloramine solution at room temperature for disinfection purposes.

**Fig 1 Fig1:**
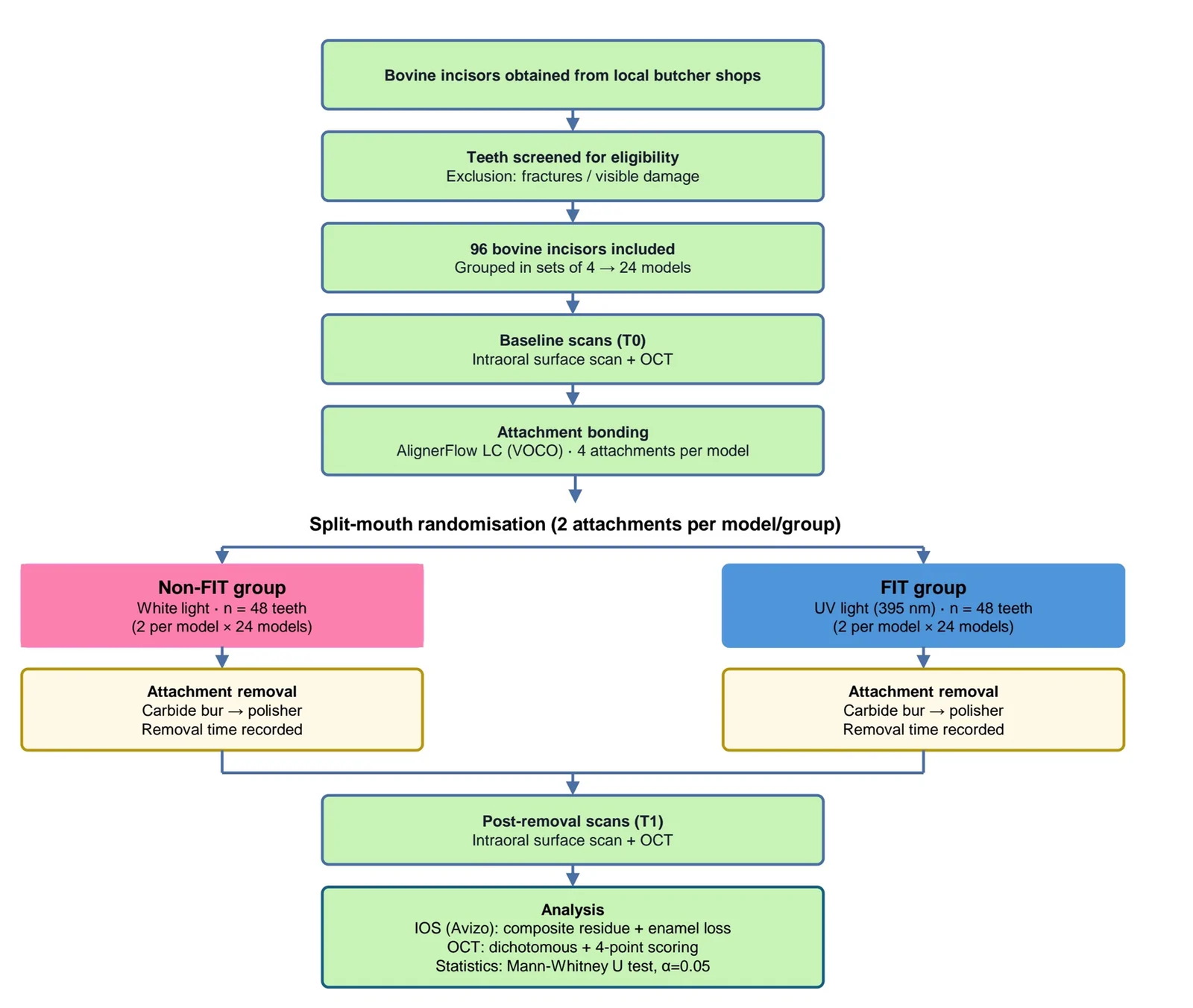
Study flowchart. Bovine incisors were screened for eligibility and allocated to 24 four-tooth models. After baseline intraoral surface scanning and optical coherence tomography (OCT), fluorescent composite attachments (AlignerFlow LC, VOCO, Cuxhaven, Germany) were bonded to all teeth. Using a split-mouth design, two teeth per model were randomly assigned to attachment removal under ultraviolet light (FIT group, 395 nm, n = 48) and two under white light (non-FIT group, n = 48). Removal time was recorded for each tooth. Post-removal surface scans and OCT were obtained and analyzed for composite residue and enamel loss using Avizo software and a semiquantitative OCT scoring system.

To standardize enamel surface areas, the labial surfaces were flattened using a diamond bur under water cooling at low speed (10,000 rpm). The prepared teeth were then grouped in sets of four matched for similar size and shape. These groups were used to create a dental arch that mimicked mandibular human incisors. For this purpose, a silicone putty material (Eurosil Max Lab Z Putty, Henry Schein, Melville, USA) and typodont models (Surco A-S, Frasaco, Tettnang, Germany) were used to create that arch. The putty allowed the removal and repositioning of individual teeth, and the arch could be mounted into dental mannequins. Twenty-four models were constructed (Fig 2a to d).

**Fig 2a to e Fig2atoe:**
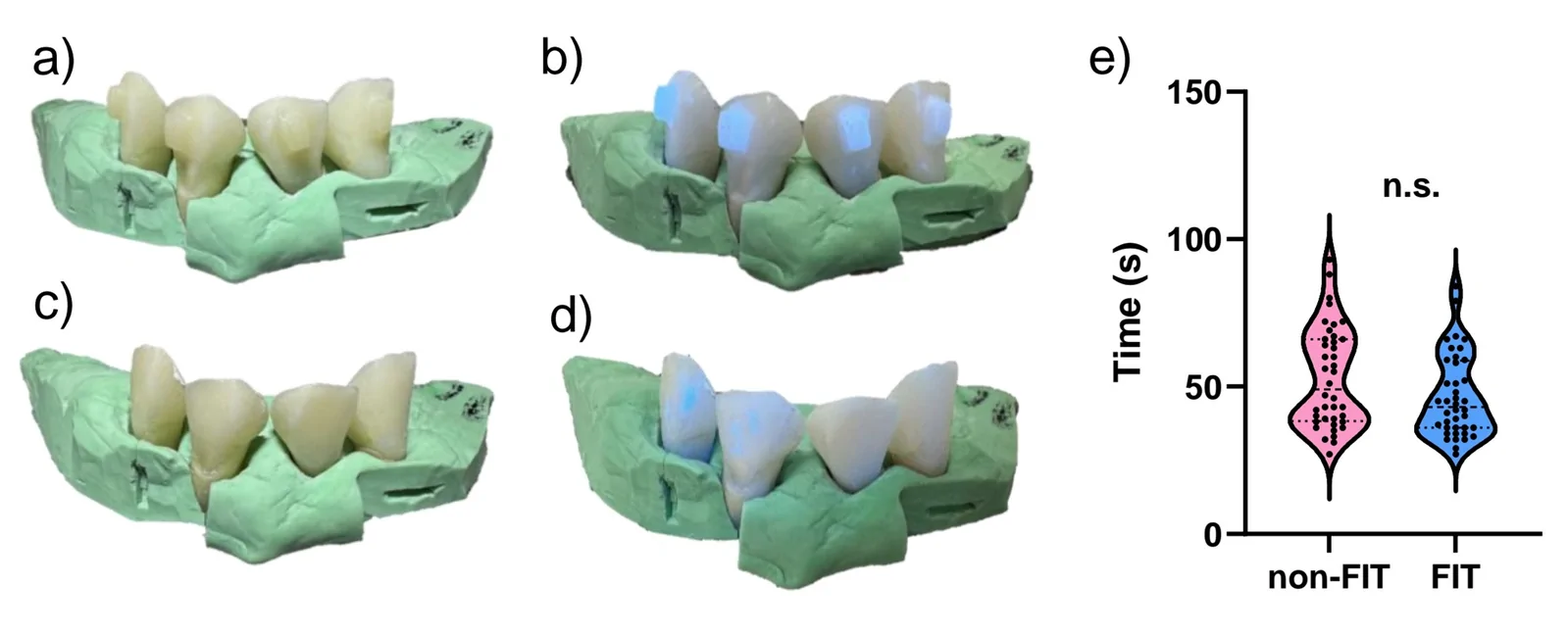
Trial setup and time measurement. (a) Bovine teeth with applied composite attachments in standard white light. (b) Bovine teeth with applied composite attachments in UV light. (c) Bovine teeth after composite removal in standard white light. (d) Bovine teeth after composite removal under UV light. Composite rests are visible on two teeth. (e) Violin plot showing the measured time required for debonding. There was no difference in removal time between non-FIT and FIT (*p* < 0.01).

Each tooth was then scanned individually with an intraoral surface scanner (TRIOS 3, 3shape, Copenhagen, Denmark) and with optical coherence tomography (SD-OCT, spectral-domain optical coherence tomography Telesto II SP2, center wavelength 1310 ± 120 nm, sensitivity ≤ 106 dB, spot size 20 µm, axial/lateral resolution < 5.5/20 μm [air], imaging speed 28 kHz, A-scan average 1, FOV 6 mm × 8 mm × 3.52 mm [pixel size 10 µm × 20 µm × 3.44 µm], Thorlabs, Dachau, Germany). The OCT scans (T0) were acquired in the area where an attachment would later be placed using the software ThorImage ver. 5.4.2 (Thorlabs, Dachau, Germany).

Afterward, dental arches were scanned, and a rectangular box attachment was virtually designed (OnyxCeph ver. 3.2.64, Image Instruments, Chemnitz, Germany). The rectangular attachments had a maximum horizontal extension of 2 mm and a vertical extension of 3 mm with a height ranging from 0.5 mm to 1 mm depending on the tooth’s anatomy. The models were then 3D-printed (ASIGA Imprimo, SCHEU-Dental, Iserlohn, Germany) using an epoxy resin (Imprimo LC Model Ivory, 385 nm, 0.05 mm thickness).

These printed models were used to produce attachment templates made from thermoformed elastic foils (COPYPLAST, Biostar/Ministar, clear, 1,0 × 125 mm, Art.- No.: 3179/1, Ch.-No. 03/4, SCHEU-Dental, Iserlohn, Germany).

### Attachment Application

To simulate clinical conditions, the models were secured in a dental mannequin (PK-2, Frasaco, Tettnang, Germany) on a dental chair (Sirona, Charlotte, NC, USA).

The enamel was etched with 33% phosphoric acid gel (Kaniedenta, Herford, Germany) for 30 s, rinsed with water for at least 10 s, and then air-dried. Orthodontic adhesive (TransBond XT, 3M, Saint Paul, USA) was applied using a microbrush, gently air-thinned, and light-cured for 20 s using a high-intensity light-curing unit (Bluephase 20i, Ivoclar, USA) (output intensity: 2000 mW/cm^2^).

For attachment application, template reservoirs were filled with a low-viscosity composite (AlignerFlow LC, VOCO, Cuxhaven, Germany) and positioned over the dental arches. Attachments were pre-cured for 5 s each, the template was removed, and a final curing step was performed for 20 s. Any obvious composite excess was removed with a scaler.

### Attachment Removal

Two attachments per model were removed under a conventional white light source of the dental chair with non-fluorescent lighting (NF), while the other two attachments were removed using a headlamp emitting UV light at 395 nm (FIT). The allocation of removal method to specific tooth positions was randomized to minimize positional bias.

Two operators, both right-handed, performed the removal procedures. Both operators were instructed to remove each attachment individually until no composite residue could be identified. The only tools used for detection were a mirror and a dental probe; magnification was not allowed.

Attachment removal was performed in two sequential steps. First, a carbide burr (123-604-30, Dentaurum, Ispringen, Germany) was used at 40,000 rpm with air cooling. Subsequently, surfaces were smoothed using a polisher (900-2105, Henry Schein, Melville, NY, USA) at 12,000 rpm with water cooling. The duration of each removal procedure was recorded, finishing once the operator was satisfied with the results. The burrs and polishing instruments were replaced after every eight teeth.

### Surface Analysis

After attachment removal, a second series of intraoral surface scans and tooth material scans by OCT were performed (T1). The baseline (T0) and post-removal (T1) intraoral surface scans of each tooth were converted into volumetric datasets and superimposed (Avizo ver. 8.1.1, ThermoFisherScientific, Darmstadt, Germany). The labial surface of the crown was manually segmented, and all surface alterations were identified. From these overlays, the volume of residual composite (T1–T0) and the volume of enamel loss (T0–T1) were quantified in mm^3^ (Fig 3a to d).

**Fig 3a to f Fig3atof:**
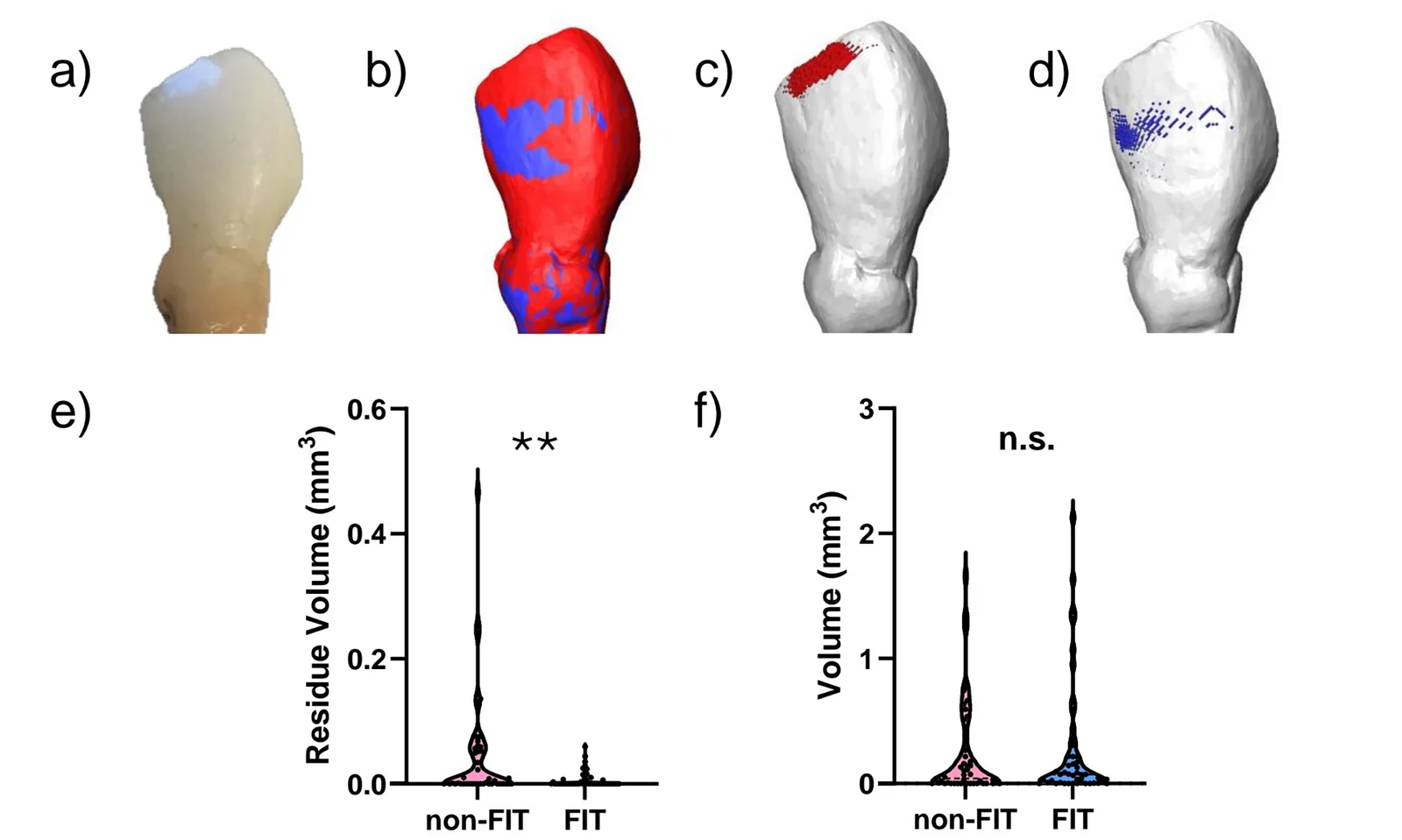
Quantification of composite residue and enamel loss on intraoral scans. (a0 Bovine tooth with composite residue visible under UV light. (b) Superimposition of intraoral scan before attachment bonding (T0, blue) and after attachment removal (T1, red). (c) Subtraction of the T1 scan from the T0 scan. The red area shows the adhesive remnants corresponding to the visible residue shown in A. (d) Subtraction of the T0 scan from the T1 scan. The blue area shows the region of enamel loss. (e) Violin plot showing a comparison of the volume of residue left. There is significantly less residue when using FIT (*p* < 0.01). (f) Violin plot showing a comparison of the volume of enamel loss. No significant differences between the groups.

For OCT analysis, the custom-made molds were used to ensure precise repositioning of the teeth (T1). 300 separate pictures were exported (OCTex View ver. 1.0.3). The T0 and T1 image stacks were then manually aligned in ImageJ, using surface contours and characteristic features such as enamel cracks as reference points (Fig 4a and b).

**Fig 4a to d Fig4atod:**
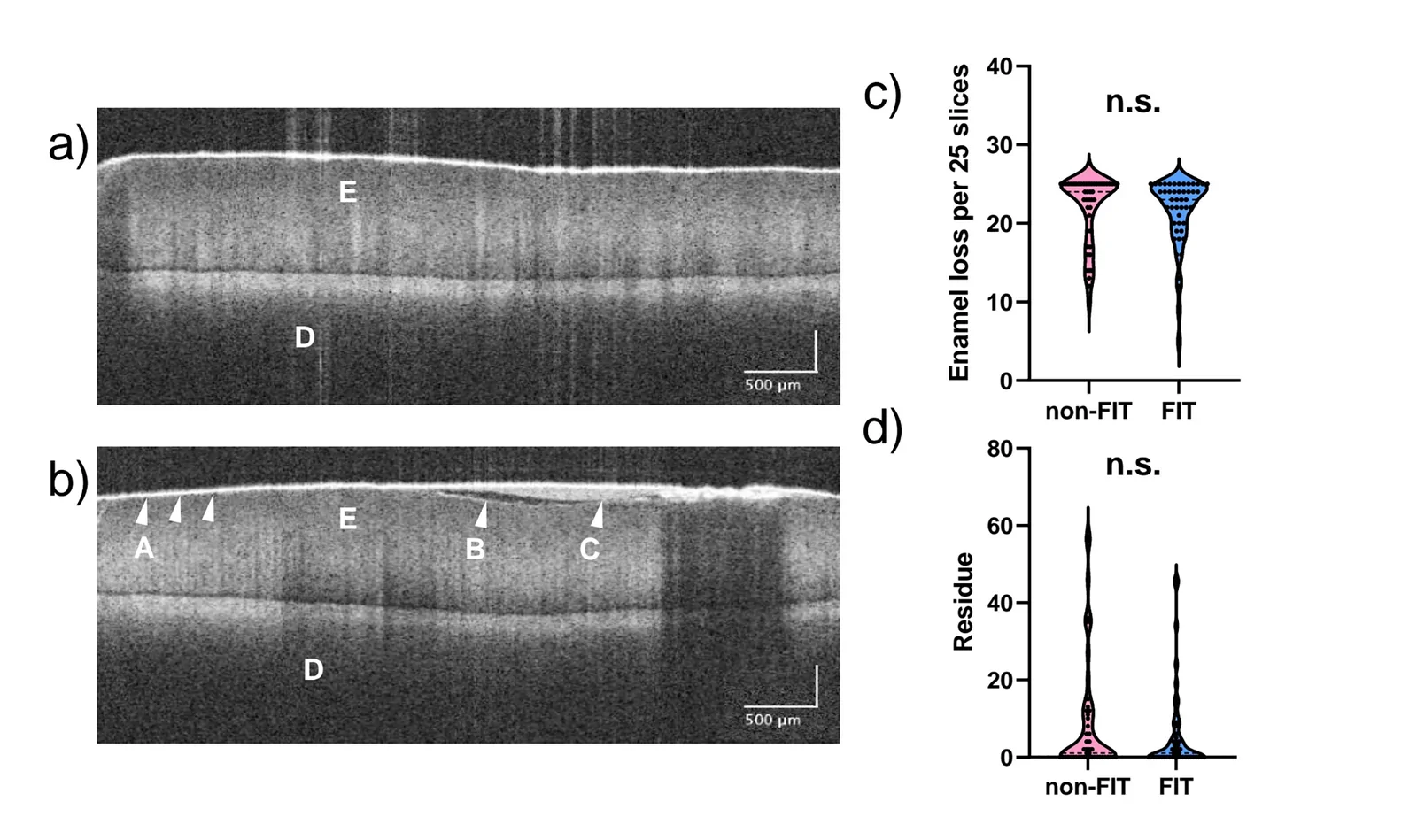
OCT assessment. (a) A slice of an OCT scan of a bovine tooth before composite bonding (T0). (b) The same slice of the same tooth after composite bonding and removal (T1). Enamel (E), dentine (D), abrasion (A), bonding remnants (B), and composite remnants (C) are shown. (c) Violin plots showing quantification of enamel loss per 25 OCT-slices. The abrasion was quantified with a dichotomous scoring system. (d) Violin plots showing quantification of composite residue per 25 OCT slices. The residue was quantified with a four-point scoring system. No significant differences between the groups.

After alignment, 25 equidistant slices were generated for evaluation.30 Enamel loss was assessed using a dichotomous scoring system (0 = no enamel loss; 1 = visible abrasion). For each specimen, the number of affected slices was summed across all 25 slices, leading to a maximum possible score of 25. Composite residues were categorized with a four-point scoring system: no residue (score 0), adhesive remnants only (score 1), composite residue only (score 2), or both adhesive and composite residue (score 3). The four-grade scoring system is a simplified version of the six-grade scoring system used to describe the adhesive residues used for fixed appliances (brackets) in human molars introduced by Park et al (2025).^25^ To account for both the prevalence and severity of residue, a weighted sum scale was calculated by multiplying the number of slices assigned to each score by its score value. This leads to a maximum score of 75.

### Statistical Analysis

To evaluate any differences between the non-FIT and FIT group, statistical analyses were performed using GraphPad Prism (GraphPad Prism ver. 10.4.1., GraphPad, Boston, USA) with a significance level of α = 0.05. Prior to group comparisons, data were tested for normal distribution using a Shapiro–Wilk test. Data for debonding times, enamel loss and composite residue were not normally distributed (Shapiro–Wilk test, *p* < 0.05), so group differences were assessed using the Mann–Whitney U test. All tests were two-tailed.

To assess scoring reliability, ten specimens were randomly selected and rescored by the primary examiner after a 3-week interval (intra-observer reliability) and independently by a second examiner familiar with the OCT method (inter-observer reliability). Cohen’s kappa (κ) was calculated for the dichotomous enamel loss criteria (GraphPad Prism ver. 10.4.1., GraphPad, Boston, MA, USA), and quadratically weighted Cohen’s kappa (κw) for the ordinal composite residue score (0–3) (R version 4.5.2, Foundation for Statistical Computing, Vienna, Austria), across all 250 slices. Intra-observer reliability was almost perfect for both the enamel loss criteria (κ = 0.828, 94.4% agreement) and the composite residue score (κw = 0.904, 98.0% agreement). Inter-observer reliability was moderate for enamel loss (κ = 0.467, 82.4% agreement) and fair for the composite residue score (κw = 0.252, 88.8% agreement).

## Results

### Removal Time

Mean removal time of non-FIT attachments was 52.95 s, whereas the mean removal time of FIT attachments was 46.52 s, showing slightly faster removal times for FIT (Fig 2e). However, this difference did not reach statistical significance (Mann–Whitney U = 979.0, *p* = 0.086).

### Composite Residue and Enamel Loss

Composite residue and enamel loss were assessed on intraoral surface scans using Avizo software. These analyses revealed a mean composite residue volume of 0.043mm^3^ in the non-FIT group and 0.00653mm^3^ in the FIT group, corresponding to an 84.8% reduction in composite residue with FIT. This difference was statistically significant (*p* = 0.0068, Fig 3e).

Analysis of the intraoral surface scans showed mean abrasion values of 0.34mm^3^ in the non-FIT group and 0.28mm^3^ in the FIT group, corresponding to a 16.03% increase in enamel abrasion with FIT (Fig 3f), However, this difference did not reach statistical significance due to high interindividual variance in both groups (*p* value = 0.87). To further evaluate composite residue, the surface was analyzed using optical coherence tomography (OCT). Comparing a bovine tooth before (Fig 4a) and after (Fig 4b) composite bonding and attachment removal revealed the presence of residual bonding material and composite remnants.

Quantification of the OCT scans revealed a mean weighted composite residue score of 5.75 (SD 10.88) in the FIT group and 9.15 (SD 15.35) in the non-FIT group, with no statistically significant difference between the groups (Mann–Whitney U = 1042.5, *p* = 0.626, Fig 4d).

OCT analysis of enamel defects revealed enamel abrasion in 21.35 out of 25 slices in the FIT group and 22.09 slices in the non-FIT group (Mann–Whitney U = 896.5,* p* = 0.106, Fig 4c). This indicates that enamel abrasion is present in most of the specimens, regardless of whether FIT is used, with no statistically significant difference between the groups.

## Discussion

The present study demonstrates that removing attachments using FIT significantly reduces the amount of composite residue. However, as neither enamel loss nor removal time were affected, the study suggests that FIT primarily improves the quality of attachment removal rather than its efficiency. Although many studies have demonstrated that using FIT can reduce the time needed for debonding fixed appliances,^2,3,23
^ this is the first study to analyze its use for removing clear aligner attachments. One possible explanation for why FIT did not reduce the removal time is related to the clinical accessibility of the attachments. In this study, all attachments were located on the vestibular tooth surface, allowing unrestricted access during removal. Previous work highlighted the advantages of the method, particularly in areas that are difficult to access.^7^ However, in a previous study analyzing the use of FIT for removing orthodontic retainers, there was observed only a non-significant tendency toward faster removal with FIT.^34^ As retainer adhesive sites are typically less accessible than vestibular attachments, this may indicate that the time-saving effects of FIT are subtle and highly dependent on the clinical conditions.^34^ Indeed, the variability in removal times was very high in our study, which may explain why the decrease in removal times did not reach statistical significance.

In line with this observation is a study showing that the differences in debonding time were strongly influenced by the individual operator, with some clinicians achieving faster removal times than others.^24^ Consequently, positive effects of FIT may be masked by operator-related variability.^24^ Accordingly, another study also observed no evidence of a time-saving effect of FIT.^13^ To clarify whether FIT may save time during debonding procedures, future research should include a larger number of clinical operators and a stratified analysis comparing experienced and inexperienced clinicians, while also considering different visibility conditions during resin removal. In this context, a recent study demonstrated that the use of dental loupes reduced composite remnants and enamel loss after clear aligner attachment removal, suggesting that the combination of magnification and fluorescence-aided identification represents a promising avenue for future investigation.^38^


In clinical practice, however, the time of attachment removal is not the only thing to consider when choosing an appropriate composite material. The amount of residual material and the integrity of the enamel surface are equally important. Therefore, composite residue and enamel surface integrity were subsequently assessed using different imaging techniques. To evaluate the surface after debonding, pre- and post-treatment optical scans were aligned and volumetrically compared. Although the accuracy of intraoral surface scanners is influenced by factors such as surface conditions and handling,^4,26,27
^ the surface scanner that was used in the present study has previously demonstrated high volumetric precision compared with similar systems.^21^ Based on the analysis of intraoral surface scans, the use of FIT resulted in significantly less residual composite, which is consistent with findings from previously published studies.^7,8,16,28
^


In contrast, OCT measurement did not reveal significant differences in the presence of residual composite between FIT and non-FIT. However, this does not contradict the volumetric findings, as OCT allows the detection of whether remnants are present within individual cross sections, but does not permit a quantitative assessment of their volume. Additionally, here a very simplified scoring system was used, which has *per se* a coarser scale as compared to the continuous scale of the surface measurements. However, both methods demonstrate that composite residues occur regardless of the composite material that was used, and volumetric surface analysis shows that the amount of residual material can be significantly reduced using FIT.

While both removal methods lead to residual composite in differing amounts, this naturally leads to the question of to what extent these residues influence the enamel integrity. The OCT measurements show that small defects in the enamel surface were visible in nearly all sections regardless of the used composite material. These findings suggest that small enamel defects are a foreseeable consequence of attachment removal rather than a method–dependent phenomenon.^6,11,35
^ Additionally, the OCT measurements are characterized by high spatial resolution and can detect even subtle surface changes, giving a remarkably high sensitivity.^10^ Moreover, the manual scoring system used for OCT images revealed inter- and intra-operator variability, indicating that an automated approach could improve the robustness of future research. Previous studies have demonstrated reduced enamel defects associated with the use of FIT compared to conventional methods.^3,7
^ However, it should be noted that in this study highly sensitive OCT measurements were not applied, which may limit direct comparability with the present findings.^25^ Taken together, the present findings indicate that FIT does not increase the risk of enamel damage compared with conventional methods.

A limitation of this study lies in the use of bovine teeth instead of human teeth. In consequence of the increased prevalence of conservative dental interventions, most of the extracted human teeth available for analysis are characterized by signs of decay or previous restorative treatments, making them unsuitable for utilization in this study. Bovine teeth, in contrast, are characterized by their ready availability, minimal decay, and flat surfaces that are ideal for the application of orthodontic attachments. Nevertheless, they are accepted as a substitute for human teeth due to their similar chemical composition,^5^ microstructure,^39^ and luminescence^32^ to human enamel.^31^


Another limitation is that a clinical environment cannot fully be replicated *in vitro*, as factors such as saliva and movement are absent, which can influence debonding behavior under clinical conditions. However, the controlled experimental setting allowed for standardized comparison of debonding techniques and strengthened internal validity. In addition, although fluorescent properties of materials may change over time,^14,36
^ the typical duration of aligner therapy is approximately 12 months, suggesting that these effects can be neglected in the present study.

## Conclusion

FIT significantly reduced residual composite after attachment removal while neither

debonding time nor enamel loss were affected. Future *in vivo* studies with larger

sample sizes, multiple operators and different adhesive systems are needed to validate

these findings.

### Acknowledgments

Language refinement was assisted using DeepL Translate and DeepL Write (DeepL SE, Cologne, Germany; 2025, https://www.deepl.com) to enhance clarity and style. Content and interpretations, however, are original and produced solely by the authors.

## References
